# Brain Microstructural Abnormalities Are Related to Physiological Alterations in End-Stage Renal Disease

**DOI:** 10.1371/journal.pone.0155902

**Published:** 2016-05-26

**Authors:** Zhigang Bai, Xiaofen Ma, Junzhang Tian, Jianwei Dong, Jinlong He, Wenfeng Zhan, Lijuan Xu, Yikai Xu, Guihua Jiang

**Affiliations:** 1 Department of Medical Imaging Center, Nanfang Hospital, Southern Medial University, Guangzhou City, Guangdong province, PR China; 2 Department of Medical Imaging, Guangdong No. 2 Provincial People’s Hospital, Guangzhou City, Guangdong province, PR China; 3 Department of Mathematics, Guangdong Pharmaceutical University, Guangzhou City, Guangdong province, PR China; 4 Image diagnostics division, the Affiliated Hospital of Inner Mongolia Medical University, Huhehaote City, Inner Mongolia Autonomous Region, PR China; 5 National Laboratory of Pattern Recognition, Institute of Automation, Chinese Academy of Sciences, Beijing, PR China; Hospital Universitario de La Princesa, SPAIN

## Abstract

**Purpose:**

To study whole-brain microstructural alterations in patients with end-stage renal disease (ESRD) and examine the relationship between brain microstructure and physiological indictors in the disease.

**Materials and Methods:**

Diffusion tensor imaging data were collected from 35 patients with ESRD (28 men, 18–61 years) and 40 age- and gender-matched healthy controls (HCs, 32 men, 22–58 years). A voxel-wise analysis was then used to identify microstructural alterations over the whole brain in the ESRD patients compared with the HCs. Multiple biochemical measures of renal metabolin, vascular risk factors, general cognitive ability and dialysis duration were correlated with microstructural integrity for the patients.

**Results:**

Compared to the HCs, the ESRD patients exhibited disrupted microstructural integrity in not only white matter (WM) but also gray matter (GM) regions, as characterized by decreased fractional anisotropy (FA) and increased mean diffusivity (MD), axial diffusivity (AD) and radial diffusivity (RD). Further correlation analyses revealed that the in MD, AD and RD values showed significantly positive correlations with the blood urea nitrogen in the left superior temporal gyrus and significantly negative correlations with the calcium levels in the left superior frontal gyrus (orbital part) in the patients.

**Conclusion:**

Our findings suggest that ESRD is associated with widespread diffusion abnormalities in both WM and GM regions in the brain, and microstructural integrity of several GM regions are related to biochemical alterations in the disease.

## Introduction

End-stage renal disease (ESRD) is a disease characterized by multi-organ dysfunction. It typically occurs when chronic renal failure progresses to a point where the kidneys are permanently functioning at less than 10% of their capacity [1]. ESRD is frequently accompanied by serious neurological problems (e.g., uremicencephalopathy, hypertensive encephalopathy and osmotic demyelination syndrome), as evidenced by focal white matter (WM) lesions and common demyelination in the brain [2–4]. The ESRD-related neurologic complications significantly worsen the prognosis and increase mortality of the disease [2,5–8]. Thus, a comprehensive mapping of ESRD-related brain abnormalities, particularly WM alterations, is of great significance to help early diagnosis and improve prognosis.

Diffusion tensor imaging (DTI) is a promising technique to study human brain structure, especially WM anatomy, by providing multiple quantitative parameters to characterize tissue microstructure from different aspects [9]. With this technique, Hsieh and colleagues investigated WM integrity of several prior regions of interest (ROIs) for patients with ESRD following long-term hemodialysis. They found that the ESRD group had significantly lower fractional anisotropy (FA) in all the ROIs (e.g., bilateral parietal, frontal, occipital and temporal regions and corpus callosum) than the control group [10]. Using a tract-based spatial statistics (TBSS) method, Zhang and colleagues subsequently demonstrated that the ESRD-related disruption of WM integrity was widely distributed [11]. Based on an automatic, objective and bias-free voxel-based method, Chou et al. further showed that microstructural alterations occurred not only in WM structures but also gray matter (GM) regions (e.g., cerebrum and pons) [12]. These studies jointly indicate that microstructural alterations may be a general output in the ESRD’s brain, which are thought to reflect renal dysfunction. However, little is known regarding the relationship between the brain abnormalities and physiological alterations induced by failure of renal function.

To fill this gap, we collected DTI data from 38 ESRD patients and further recorded their renal metabolin (e.g., serum creatinine, Scr, and blood urea nitrogenurea, BUN), vascular risk factors (e.g., blood pressure and cholesterol) and general cognition (i.e., the Mini-Mental State Examination, MMSE). A voxel-wise analysis was then used to identify ESRD-related microstructural alterations compared with 40 age- and gender-matched healthy controls (HCs). Finally, the relationships between brain microstructural integrity and physiological, cognitive and clinical measures were examined in the patients.

## Materials and Methods

### Participants

This study was approved by the Research Ethics Review Board of the Institute of Mental Health at the Southern Medical University, and written informed consent was obtained from each participant. A total of 48 patients with ESRD (all right-handed) were recruited from the renal transplantation department at Guangdong No. 2 Provincial People's Hospital, Guangzhou, China from August 2011 to July 2013. Exclusion criteria included: (1) psychiatric disorders or major neurologic disorders (e.g., severe head injury, stroke, epilepsy, dementia, anxiety, depression or visible lesions) according to an experienced physician (G. X., with 10-year experience in neurology); (2) ischemic diseases including acute ischemic cerebrovascular disease, acute peripheral arterial occlusion, advanced liver or heart failure; (3) asymptomatic coronary ischemia by electrocardiogram testing; (4) a history of diabetes; and (5) substance abuse including drugs, alcohol or cigarettes. Conventional MR images were examined by an experienced radiologist (W. L., with 20-year experience in neuropathology) who was blinded to whether the images were from the patient or control group. Thirteen patients were excluded due to failure of MRI scanning (10) or abnormal hyper-intensities in the T2-FLAIR MR images (3). Therefore, the final study population included 35 patients with ESRD (28 males; mean age 37.5 ± 11.3 years, range 18–61 years).

Forty age- and gender-matched HCs (all right-handed; 32 males; mean age 41.5 ± 10.6 years, range 22–58 years) were recruited from the local community. All the HCs had no physical diseases or history of psychiatric or neurologic diseases. All the HCs had normal renal function as determined by no abnormal findings in the abdominal MR imaging and normal levels of serum creatinine and BUN.

All the participants completed blood pressure, MMSE [13] and multiple biochemical tests after the hemodialysis (within 36 hours) but before the MR imaging (within 24 hours). The biochemical tests included Scr, BUN, cholesterol, hemoglobin, serum kalium and serum calcium. The serum calcium levels were corrected with serum albumin levels using the Payne's formula [14].

In this study, none of the patients were on erythropoiesis-stimulating agents and treated with vitamin D, calcitriol and/or phosphorus-chelating agents. We did not check the serum PTH level for the ESRD patients. The dialysis modality and duration were also recorded from the patients’ medical history. Out of the 35 patients, 24 (68.6%) had hypertension and 6 (17.1%) had hyperlipidemia. None of the patients have a history of diabetes.

All the demographic and clinical information are summarized in Table 1.

**Table 1:** Demographics and clinical characteristics of all participants.

	ESRD (n = 35)	HCs (n = 40)	P-value
Gender (M/F)	28/7	32/8	>0.999^a^
Age (yrs)	37.5±11.3 (18–61)	41.5±10.6 (22–58)	0.119^b^
Education level (yrs)	11.8±3.2 (3–16)	10.8±2.8 (6–18)	0.148^b^
MMSE	26.9±1.9 (22–30)	29.5±0.9 (26–30)	<0.001^b^
Dialysis duration (mths)	15.5±6.6 (6–30)	-	-
Blood systolic pressure	158.5±19.5 (120–190)	102.2±8.8 (90–120)	<0.001^b^
Blood diastolic pressure	90.0±11.1 (60–110)	70±6.4 (60–80)	<0.001^b^
Serum calcium (mmol/L)^c^	2.3±0.2 (1.9–2.9)	2.3±0.2 (2.1–2.7)	0.535^b^
Serum kalium (mmol/L)^c^	4.5±0.9 (2.9–6.3)	4.3±1.0 (2.2–6.3)	0.317^b^
Hemoglobin (g/L)^c^	102.5±23.5 (56–158)	107.3±9.7 (88–124)	0.357^b^
Serum creatinine (μmol/L)^c^	838.1±483.6 (80–2030)	168.7.1±213.8 (58–920)	<0.001^b^
Blood urea nitrogenurea (mmol/L)^c^	17.8±8.2 (4.1–30.2)	5.6±1.4 (2.8–7.3)	<0.001^b^
Cholesterol (mmol/L)	5.1±1.4 (3.7–9.9)	3.9±0.4 (3.1–4.8)	<0.001^b^

Values are represented as mean±SD (min-max). ESRD, end-stage renal disease; HCs, healthy controls.

^a^The P-value was obtained by a chi-square test.

^b^The P-values were obtained by two-side two-sample t-tests.

^c^Data were missing for six patients.

### Data acquisition

All participants were scanned using a 1.5-T MR scanner (Achieva Nova-Dual; Philips, Best, the Netherlands) at the Department of Medical Imaging, Guangdong No. 2 Provincial People’s Hospital. The conventional imaging sequences, which included T1-weighted images and T2-FLAIR images, were obtained for each participant to detect clinically silent lesions. DTI data were acquired in 32 diffusion gradient directions (b = 800 s/mm^2^ along 32 non-collinear directions) plus a reference image (i.e., b = 0) using a single shot spin echo planar sequence. The parameters were as follows: TR = 10,793 ms, TE = 62 ms, field of view = 230×230 mm^2^, matrix = 128×128, slice thickness = 2 mm, no slice gap, voxel size = 2×2×2 mm^3^. For each participant, resting-state fMRI data and T1-weighted three-dimensional data were also acquired which were not used in the current study.

### Diffusion image processing

Diffusion images were processed using the PANDA toolbox [14] (http://www.nitrc.org/projects/panda/) based on the FSL. First, individual diffusion-weighted images were co-registered to their corresponding b0 images using an affine transformation to correct for head motion and eddy current-induced distortions. The resultant affine transformations were further used to rotate gradient directions of each diffusion image. Then, the diffusion tensors were estimated by solving the Stejskal and Tanner equation [15,16] and the reconstructed tensor matrix was diagonalized to obtain 3 eigenvalues (λ_1_, λ_2_, λ_3_) and their corresponding eigenvectors. The FA, mean diffusivity (MD), axial diffusivity (AD) and radial diffusivity (RD) were subsequently calculated for each voxel as:

$$FA=\frac{\sqrt{{\left(\lambda 1-\lambda 2\right)}^{2}+{\left(\lambda 1-\lambda 3\right)}^{2}+{\left(\lambda 2-\lambda 3\right)}^{2}}}{\sqrt{2(\lambda 1^{2}+\lambda 2^{2}+\lambda 3^{2})}}$$ (1)

$$MD+\frac{\lambda 1+\lambda 2+\lambda 3}{3}$$ (2)

AD=λ1 (3)

$$RD=\frac{\lambda 2+\lambda 3}{2}$$ (4)

Finally, the resultant FA, MD, AD and RD maps were spatially normalized into the Montreal Neurological Institute (MNI) space to promote group analysis. Specifically, the spatial normalization was performed by warping individual FA maps to the FA template provided in the FSL toolbox using a non-linear registration procedure (fsl command, fnirt). The resultant transformation matrices were then applied to the MD, AD and RD maps. All the normalized maps (FA, MD, RD and AD) were resampled into a 2×2×2 mm^3^ resolution. To ameliorate residual misalignments during spatial normalization and improve the validity of subsequent statistical inferences, we also conducted a spatial smoothing for the normalized FA, MD, RD and AD maps using a 6-mm (i.e., three times of the voxel size) full width at half maximum Gaussian kernel.

### Statistical analysis

Two sample t-tests were performed to compare the FA, MD, AD and RD maps between the ESRD and HCs groups. To minimize potential effects of head motion on our results, we calculated a total motion index (TMI) for each participant and treated individual TMI values as a covariate during the between-group comparisons [17]. To estimate the relationship between brain microstructure and physiological indictors in the ESRD patients, we performed a correlation analysis between each DTI metric (FA, MD, RD and AD) and each of the Blood pressure, Serum calcium, Serum kalium, Scr, BUN, Cholesterol, hemodialysis duration and MMSE score. All the correlation analyses were conducted in a voxel-wise manner over the whole brain to avoid a circular analysis[18]. For both between-group comparison and correlation analyses, the results were presented at the statistical threshold of *p* < 0.05 (corrected) by combined a height threshold of *p* < 0.001 [19] for individual voxels and an extent threshold of *p <* 0.05, as determined by Monte Carlo simulations[20].

## Results

### Demographic, biochemical and clinical characteristics

The demographic, biochemical and clinical characteristics for all the participants are shown in Table 1. There were no significant differences in gender (*p >* 0.999), age (*p* = 0.119), education level (*p* = 0.148), serum calcium (*p* = 0.535), serum kalium (*p* = 0.317) or hemoglobin (*p* = 0.357) between the ESRD and HC groups. Compared with the HCs, the patients with ESRD exhibited significantly lower MMSE scores and significantly higher blood systolic pressure, blood diastolic pressure, serum creatinine, cholesterol and BUN (all *p* < 0.001).

### Between-group differences in FA

**Fig 1:**
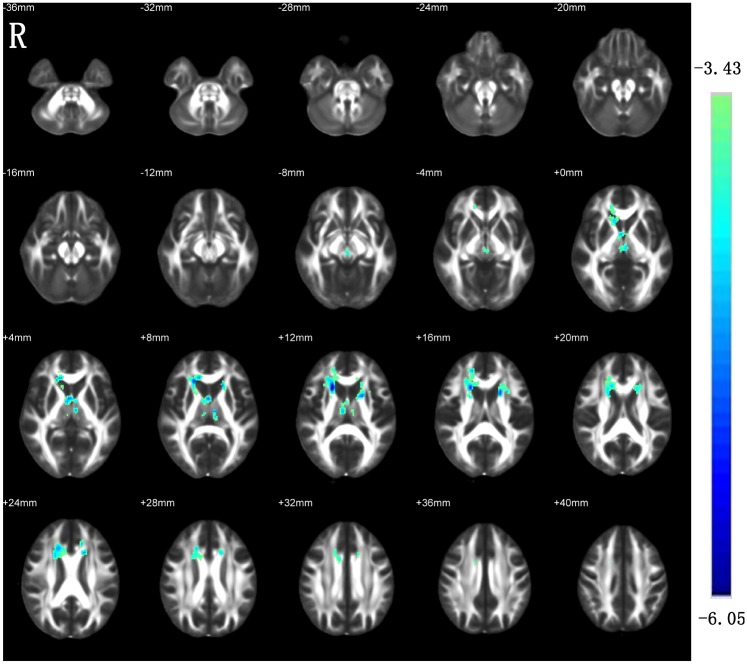
Decreased FA in the patients with ESRD as compared with HCs. R, right.

Compared with the HCs, the ESRD patients showed significantly decreased FA in the bilateral anterior limb of internal capsule (ALIC), fornix, genu of corpus callosum and anterior commissure (Fig 1).

### Between-group differences in MD

**Fig 2:**
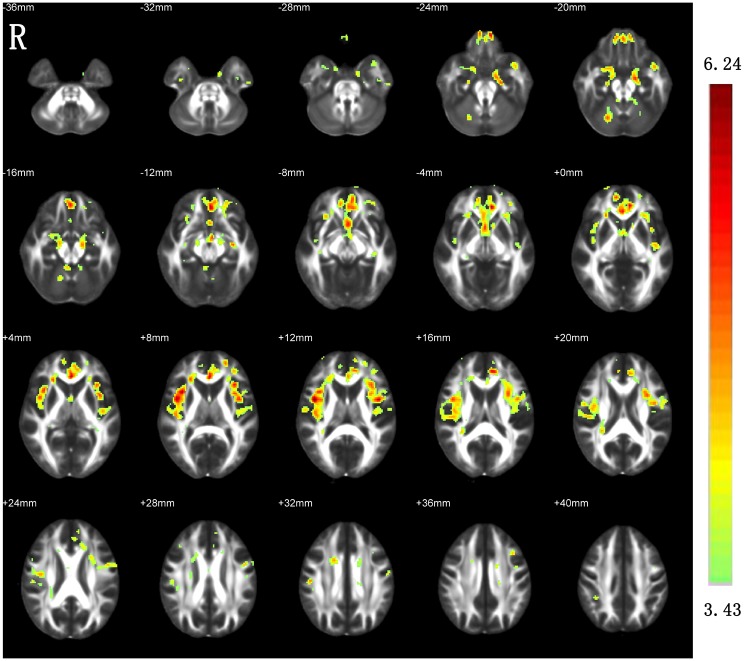
Increased MD in the patients with ESRD as compared with HCs. R, right.

Compared with the HCs, the ESRD patients showed significantly increased MD in the bilateral hippocampus, parahippocampal gyrus, amygdala, insula, ALIC, anterior cingulate cortex, corpus callosum, medial prefrontal cortex, superior temporal gyrus, fornix and anterior commissure, and the right cerebellum (Fig 2).

### Between-group differences in AD and RD

**Fig 3:**
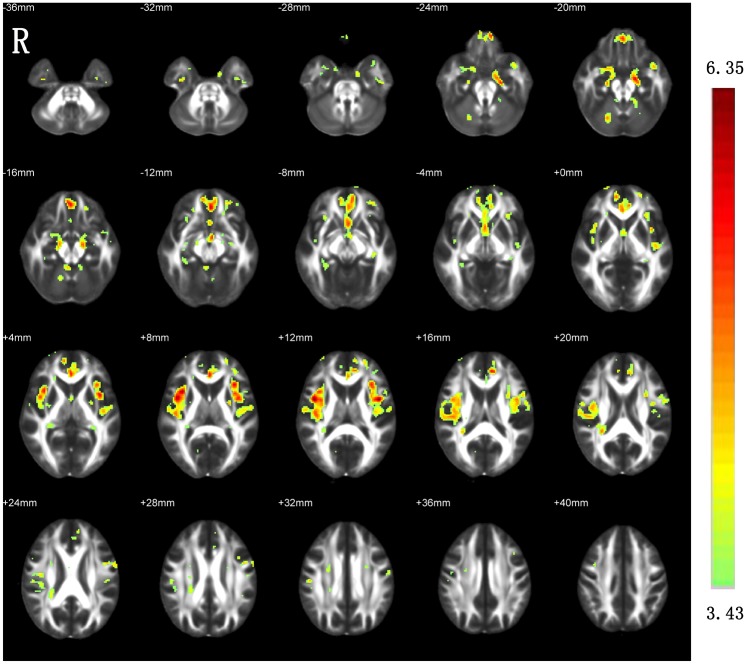
Increased AD in the patients with ESRD as compared with HCs. R, right.

**Fig 4:**
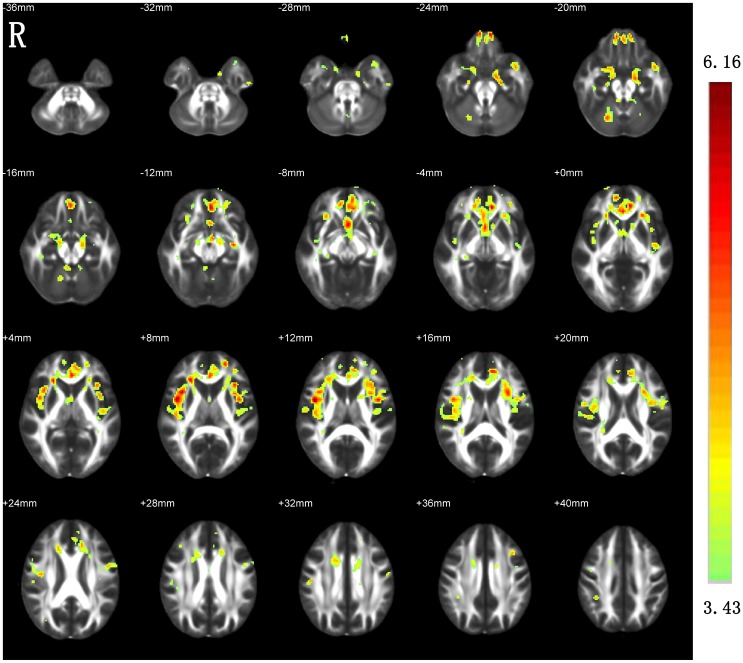
Increased RD in the patients with ESRD as compared with HCs. R, right.

The between-group differences in AD (Fig 3) and RD (Fig 4) were very similar to those revealed by MD.

### DTI-clinical relationship

**Fig 5:**
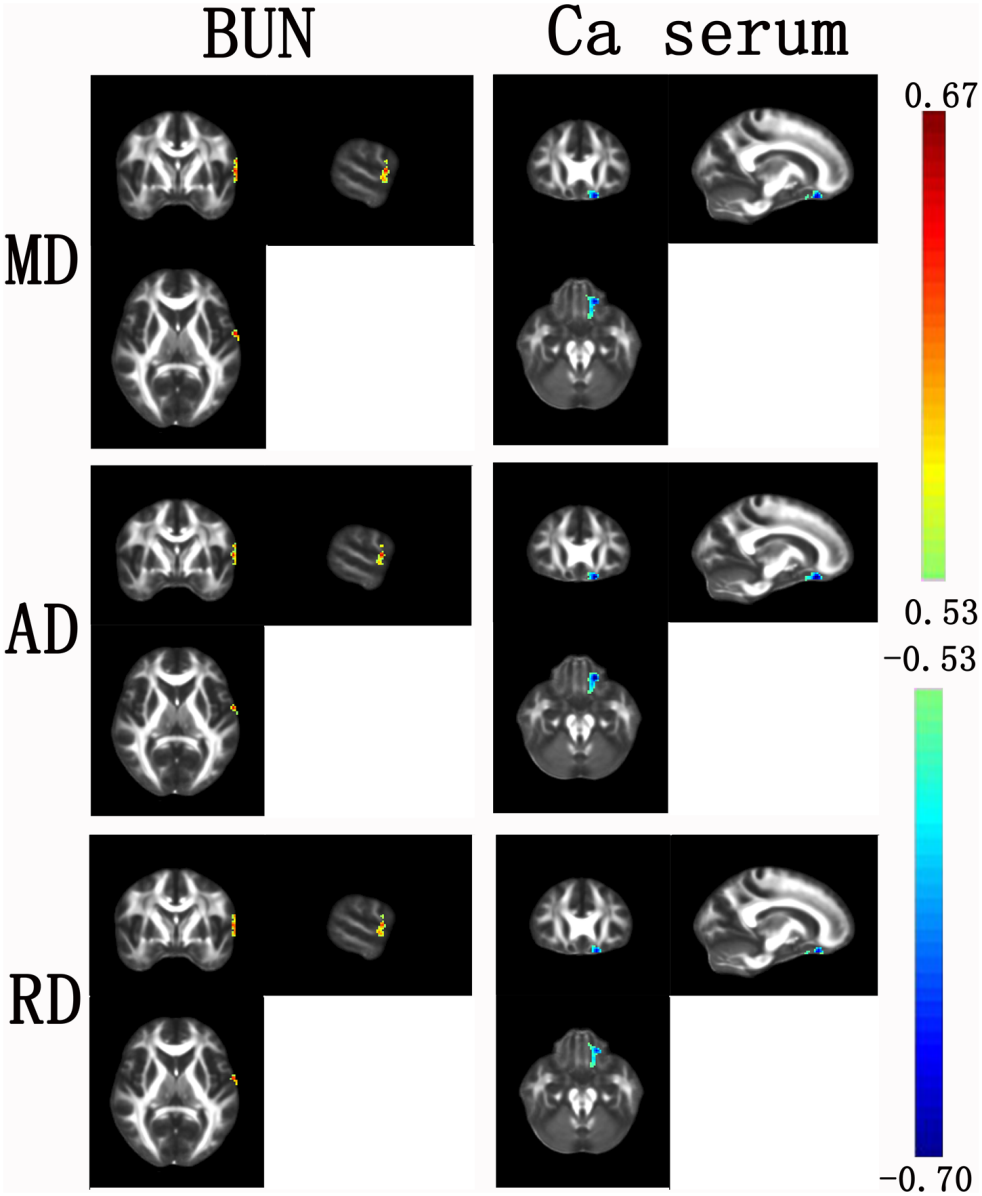
The relationships between DTI metrics and biochemical indictors in the ESRD patients. R, right.

There were no regions showing significant correlations between any clinical index and the FA values. As for MD, a significantly negative correlation was observed with the corrected calcium levels in the left superior frontal gyrus (orbital part) and a significantly positive correlation was observed with BUN in the left superior temporal gyrus. Again, the AD and RD exhibited almost the same correlation patterns to those revealed by MD (Fig 5).

## Discussion

In this study, we used a voxel-wise analysis method to study whole-brain microstructural alterations in patients with ESRD and further examined the relationship between brain microstructure and physiological indicators in the disease. Our results showed widespread microstructural alterations in ESRD that involved not only WM but also GM regions. Moreover, microstructural features of several GM regions were related to physiological indices in the patients. These findings may help understand the interaction between brain abnormalities and physiological alterations in ESRD.

We found that several WM regions showed decreased FA in the ESRD patients as compared with the HCs, including the bilateral ALIC, fornix, genu of corpus callosum and anterior commissure. These findings are largely consistent with previous DTI studies in ESRD [12,21,22]. The decreased FA indicates disrupted microstructure integrity in these regions, which could be due to axonal degeneration or demyelination induced by ESRD. Specifically, the ALIC is involved in two important circuits: the medial circuit, composed of the hippocampal formation, mammillary bodies, anterior thalamic nuclei and cingulate gyrus, and the basolateral limbic circuit, composed of the orbitofrontal cortex, dorsomedial thalamic nucleus, amygdala and anterior temporal cortex [23–25]. Previous studies have found that disruptions of structural and/or functional connectivity of components in these two circuits are related to multiple psychological disorders, such as depression [26]. Thus, we speculate that the decreased FA in the ALIC could be a sign for the possible development of psychological disorders in ESRD. However, there is another possibility that the current patients may be complicated by depression, which further leads to the observed FA decrease in the ALIC. Future studies are needed to clarify this issue.

In addition to decreased FA, we found that the bilateral ALIC, fornix, corpus callosum and anterior commissure also exhibited increased MD, AD and RD in the ESRD patients. This is also comparable with previous studies [12,21]. These three metrics reflect the overall or direction-specific capability of tissue water diffusion. It typically increases when brain regions experience an unpredictable combination of demyelination, axon loss, gliosis and inflammation [27]. Thus, the observed increases may reflect decrease of membrane density and/or increase of extracellular volume caused by axon and myelin loss [2,6,28–30]. Apart from these WM regions, several GM regions were also identified to show increased MD/AD/RD in the ESRD patients as compared with the HCs, such as the bilateral hippocampus, parahippocampal gyrus and insula and the right cerebellum. These results are partly similar to findings from a previous study [11]. Several studies have indicated that increased diffusion capability is described secondary to gliosis [31] or neuronal loss[13]. Thus, we speculate that these increases in GM regions may be attributed to gross tissue loss or occurrence of gliosis [32,33]. In addition, higher MD/AD/RD values were also found in several limbic system components, such as the bilateral amygdala and anterior cingulate cortex. Limbic system dysfunction plays a major role in numerous neuropsychiatric diseases [34], particularly in individuals with depression and anxiety [35,36]. As for ESRD, it is typically accompanied by various psychological problems [37]. For example, depression is one of the most common complications in ESRD with a prevalence rate as high as 20% to 25% [38]. Thus, we speculate that these limbic alterations may partly contribute to the prevalence of various psychological problems in ESRD, although the causal relationship should be further detailed in future. Of note, GM alterations were mainly detected by MD/AD/RD rather than FA, indicating these metrics as more sensitive biomarkers to capture ESRD-related microstructural abnormalities.

We found significantly positive correlations between the BUN and MD/AD/RD values in the left superior temporal gyrus for the patients. Evidence from neuropathologic studies indicates that long-term failure of renal function could elevate the accumulation of metabolin (e.g., BUN and Scr) and vascular risk factors (e.g., blood pressure and cholesterol) [39–41]. Galons and colleagues show that urea gradient changes could lead to increased osmotic gradient between the plasma and the interstitial or cerebrospinal fluids, which could further result in cerebral edema (e.g., intracellular/cytotoxic edema and extracellular/osmotic edema) [42,43]. Given that MD/AD/RD reflect the capability of tissue water diffusion, it is reasonable to observe positive correlations between the BUN and MD/AD/RD values because water molecules diffuse more easily under the circumstance of cerebral edema. In addition, we also found significantly negative correlations between the calcium levels and MD/AD/RD values in the left superior frontal gyrus for the patients. Previous studies have shown that dialysis patients typically present hypercalcemia, which is associated with poor mental health in the patients [44]. However, little is known about how mineral metabolism of calcium levels exerts an influence on mental health. Here, our finding may provide insights into this issue, especially considering the important role of superior frontal gyrus in cognition. It should be noted that the current study lacked cognitive measurements for the patients, thus more insights could be benefited from studies that simultaneously collect biochemical, neuroimaging and cognitive data in the same cohort of patients.

The current study has several limitations. First, the major drawback of the current study was the lack of cognitive measurements, which limited us to study the neuroanatomical significance of the observed microstructural alterations in ESRD. Second, the sample size was relatively small and the MRI scanning parameters were suboptimal (e.g., 1.5 T MRI and only one b0 image), which may limit the power in detecting more subtle effects. Future studies with a large cohort of participants and more advanced techniques and optimized parameters are needed. Third, in the current study, we used a voxel-based analysis method to find that ESRD was associated with widespread disruptions of microstructural integrity in both WM and GM regions. However, the voxel-based method may be limited by spatial misalignments and the amount of smoothing [45,46] and thus the current findings should be interpreted with caution. Future studies are required to examine the reproducibility of the current findings over different choices of registration methods and sizes of spatial smoothing. Fourth, some of the current patients suffered from hypertension and/or hyperlipidemia, which may confound our results. To provide preliminary insights into these issues, we compared the DTI metrics (FA, MD, AD and RD) between the patients with and without hypertension/hyperlipidemia. No significant differences were found in any DTI metric (*P* > 0.05, corrected). These exploratory analyses suggest limited effects of hypertension and hyperlipidemia on the current findings. Nevertheless, it should be noted that there are still other factors that may contribute to the current findings given the end stage of the disease, such as depression, a common complication in ESRD [35]. However, the current samples did not undergo neuropsychological tests for these psychological disorders. In addition, the ESRD patients in the current study received regular hemodialysis treatment, which may also affect the results, although we did not found any significant correlation between the DTI metrics (FA, MD, RD and AD) and hemodialysis duration. Future studies can help clarify these issues by using more rigorous experimental design (e.g., including neuropsychological tests) and stricter enrollment criteria (e.g., treatment-naïve ESRD patients). Fifth, the biochemical tests were performed after the hemodialysis (within 36 hours) for the patients. How to determine an “ideal” time with respect to the hemodialysis for these tests is important for the DTI-biochemical correlation analysis and different choices may affect the observed results. Finally, in the current study, we did not have the data of renal failure duration for the patients. It is an interesting topic in the future to study the relationship between ESRD-related brain alterations and renal failure duration of the patients, which could provide important insights into the origin and occurrence of brain abnormalities induced by failure of renal function.

## Conclusion

The pathophysiologic mechanism between the brain and kidney injury is complex and controversial. The current study provides novel insights into this issue by painting a comprehensive picture of brain microstructural alterations and linking the brain microstructure with biochemical indicators in ESRD.
